# IDH1 Mutant Glioma Favors Group 3 Innate Lymphoid Cells and Is Resistant to Immune Checkpoint Expression

**DOI:** 10.1007/s00011-026-02223-8

**Published:** 2026-04-01

**Authors:** Serife Erdem, Yesim Haliloglu, Inayet Nur Uslu, Mohammad Houran, Halil Ulutabanca, Alperen Vural, Mehmet Berat Erturhan, Halit Canatan, Ahmet Eken

**Affiliations:** 1 Department of Immunology, School of Medicine, Kırsehir Ahi Evran University, Kırsehir, Turkey; 2 Department of Medical Biology, School of Medicine, Erciyes University, Kayseri, Turkey; 3 Betul-Ziya Eren Genome and Stem Cell Centre, Erciyes University, Kayseri, Turkey; 4 Department of Oncology and Pathology, Karolinska Institutet, Stockholm, Sweden; 5 Department of Medical Biology, School of Medicine, Cukurova University, Adana, Turkey; 6 Department of Neurosurgery, School of Medicine, Erciyes University, Kayseri, Turkey; 7 Department of Otorhinolaryngology, Cerrahpasa Faculty of Medicine, Istanbul University - Cerrahpasa, Istanbul, Turkey; 8 Department of Immunology and Microbiology, University of Colorado Anschutz Medical Campus, Aurora, CO USA

**Keywords:** IDH-WT glioma, IDH-Mut glioma, ILC, D-2-hydroxyglutarate (D-2HG)

## Abstract

**Background:**

Isocitrate dehydrogenase 1 (IDH1) mutations confer distinct biological properties to gliomas, including the reshaping of the tumor immune microenvironment. While T cell dysfunction in glioblastoma has been extensively characterized, the role of innate lymphoid cells (ILCs)-critical regulators of tissue homeostasis and early immune responses- remains poorly understood.

**Methods:**

We investigated how IDH1 mutations and their oncometabolite D-2-hydroxyglutarate (D-2HG) influence ILC subset distribution, immune checkpoint expression, and cytokine production in glioma patients, glioma-conditioned medium (GCM) models, and in vivo mouse experiments. Tumor and peripheral blood samples from 32 glioma patients (WHO 2021 classification, grades II–IV) were analyzed by flow cytometry to assess ILC subsets and immunecheckpoint molecules (PD-1, CTLA-4, KLRG1). Tonsil-derived human ILCs were co-cultured with IDH1-mutant or wild-type glioma cells and their GCM. In vitro, ILCs were exposed to graded concentrations of D-2HG, whereas in vivo studies involved intraperitoneal administration of D-2HG or L-2HG in mice to evaluate ILC distribution across lymphoid and mucosal tissues.

**Results:**

IDH1-mutant gliomas exhibited increased ILC3 and decreased ILC1 frequencies in both tumor tissue and peripheral blood. ILC3s in IDH1-mutant tumors expressed higher PD-1, whereas ILC2s showed reduced PD-1 levels. In co-culture assays, IDH1-mutant glioma cells and their GCM suppressed PD-1 and CTLA-4 expression on ILCs while promoting proliferation. Exposure to D-2HG recapitulated these effects in a dose-dependent manner, reducing checkpoint expression and enhancing IFN-γ and TNF-α secretion. In vivo, D-2HG and L-2HG differentially altered ILC subset distribution across mucosal and lymphoid compartments.

**Conclusions:**

IDH1 mutations and their associated oncometabolite D-2HG remodel the innate lymphoid cell landscape in gliomas, driving an ILC3-biased phenotype with reduced checkpoint receptor expression. These findings identify ILCs as key modulators of glioma immunity and suggest that targeting innate immune pathways could complement existing immunotherapeutic approaches.

**Supplementary Information:**

The online version contains supplementary material available at 10.1007/s00011-026-02223-8.

## Introduction

Adult-type diffuse gliomas represent the most common primary malignant brain tumors and remain among the deadliest central nervous system (CNS) malignancies, with a median survival of approximately 12–15 months [1]. According to the 2021 World Health Organization (WHO) classification, adult-type diffuse gliomas are now categorized based on integrated histopathological and molecular features into astrocytoma, IDH-mutant (grade 2–4), oligodendroglioma, IDH-mutant and 1p/19q codeleted, and glioblastoma, IDH-wildtype (grade 4). Higher-grade gliomas are characterized by more aggressive behavior and poorer prognosis [2].

Among molecular alterations, mutations in the isocitrate dehydrogenase 1 (IDH1) gene have emerged as a defining hallmark of a subset of gliomas associated with more favorable prognosis [3]. IDH1 mutations occur predominantly in lower-grade astrocytomas and oligodendrogliomas, whereas glioblastoma, IDH-wildtype, represents the more aggressive de novo form [4–6]. The most frequent IDH1 mutation, R132H, confers a neomorphic enzyme activity that catalyzes the reduction of α-ketoglutarate (α-KG) to D-2-hydroxyglutarate (D-2HG), an oncometabolite structurally analogous to α-KG [7]. D-2HG competitively inhibits α-KG-dependent dioxygenases, including histone demethylases and TET family DNA hydroxylases, leading to widespread epigenetic reprogramming and impaired cellular differentiation [8, 9].

Although IDH1-mutant gliomas generally exhibit prolonged survival compared to IDH-wildtype counterparts, the mechanisms underlying this disparity remain incompletely understood. Accumulating evidence suggests that IDH mutations modulate the tumor immune microenvironment—either by reducing oxidative stress–induced damage or by altering immune cell infiltration and function [10].

Innate lymphoid cells (ILCs) have recently been recognized as critical components of the innate immune system. ILCs lack rearranged antigen receptors yet can rapidly produce cytokines in response to environmental cues [11]. They are classified into three major subsets—ILC1, ILC2, and ILC3—based on cytokine secretion profiles, transcription-factor dependencies, and functional parallels with T-helper cell subsets. ILC1s (including helper ILC1s and natural killer [NK] cells) resemble Th1 cells; ILC2s are analogous to Th2 cells; and ILC3s mirror Th17 cells in cytokine production. All helper ILCs are typically marked by CD127 (IL-7 receptor α) expression [12].

While ILCs have been extensively characterized at mucosal surfaces, their contribution to CNS malignancies remains largely unexplored. ILCs are detectable within the tumor microenvironment and may participate in either antitumor defense or tumor-promoting inflammation [13–15]. However, the precise distribution, phenotype, and functional properties of ILCs in gliomas—and how metabolic alterations such as D-2HG accumulation in IDH1-mutant tumors influence these cells—are poorly understood.

This study aimed to comprehensively characterize ILC subsets in glioma tissue and peripheral blood from patients stratified by IDH1 mutation status, applying the 2021 WHO classification framework. Furthermore, we utilized an isogenic U87-MG model engineered to express mutant IDH1 (R132H) to assess the impact of glioma-derived D-2HG on human ILCs in vitro. Finally, we examined the effects of exogenous D-2HG and its stereoisomer L-2HG on ILC distribution in vivo in mice. Collectively, our findings demonstrate that IDH1 mutation reshapes the ILC landscape, promoting an ILC3-dominant phenotype and modulating immune-checkpoint receptor expression, thereby providing novel insights into the immunobiology of IDH-mutant gliomas and highlighting potential innate-immune-targeted therapeutic strategies.

## Materials and methods

### Ethics approval and participants

This study was approved by the Clinical Research Ethics Committee of Erciyes University School of Medicine on January 23, 2019 (Approval No: 96681246), September 11, 2019 (Approval No: 2019/604), and December 1, 2021 (Approval No: 21/244). Tumor tissues obtained through surgical resection were histopathologically evaluated in the Department of Pathology. Patients diagnosed with diffuse gliomas classified as astrocytoma, IDH1-mutant (grade 2–4) or glioblastoma, IDH1-wildtype (grade 4), according to the 2021 WHO classification of central nervous system tumors, were included in the study. Based on pathological and molecular evaluation, patients were grouped by IDH1 mutation status (IDH1-mutant vs. IDH1-wildtype).

For the control group, peripheral blood samples (n = 20) and palatine tonsil tissues (n = 20) were obtained from individuals with no history of malignancy. Tonsil tissue was used as a control tissue for innate lymphoid cell (ILC) characterization due to its well-defined mucosal and secondary lymphoid tissue properties, enrichment in ILC subsets, and accessibility during routine tonsillectomy performed for non-malignant indications. Written informed consent was obtained from all participants. All procedures were performed in accordance with the Declaration of Helsinki and institutional ethical guidelines.

### Cell lines and reagents

Human glioma U87-MG and its isogenic IDH1 (R132H)-mutant U87-MG (#HTB14IG) cell lines were obtained from the American Type Culture Collection (ATCC). Cells were maintained in Dulbecco’s Modified Eagle Medium (DMEM) supplemented with 10% fetal bovine serum (FBS), 100 U/mL penicillin–streptomycin, and 2 mM L-glutamine at 37 °C and 5% CO₂.

D-2-hydroxyglutaric acid disodium salt (D-2HG; Sigma-Aldrich, Cat#H8378) and L-2-hydroxyglutaric acid (L-2HG; Sigma-Aldrich, Cat#SML3737) were dissolved in phosphate-buffered saline (PBS) to prepare stock solutions.

### Lymphocyte isolation and processing

Lymphocytes were isolated from glioma tissue, peripheral blood, and control tonsil samples. Tumor and tonsil tissues were mechanically dissociated and passed through a 70- µm cell strainer into sterile 50 mL tubes. Single-cell suspensions were obtained using gentle pressure with a 1 mL syringe plunger and rinsed with 1× PBS [16].

Lymphocytes were then isolated from the filtered pellet using discontinuous density gradient centrifugation with Percoll (GE Healthcare, Sweden; Cat# GE17-0891-01). Peripheral blood samples (5 mL) were collected from both patients and healthy donors following informed consent. Lymphocytes from blood were isolated using Ficoll-Paque (GE Healthcare, Sweden; Cat# GE17-1440-02), following the manufacturer’s instructions. In both protocols, lymphocytes were collected from the interface layer.

Isolated cells were cryopreserved in 10% dimethyl sulfoxide (DMSO) and stored until further use. Upon thawing, samples with low post-thaw viability or insufficient lymphocyte yield (< 1 × 10⁶ viable cells) were excluded from downstream analyses to ensure data reliability and consistency across samples. All remaining frozen samples were thawed simultaneously for staining procedures [17].

### ILC staining and flow cytometry analysis

ILC staining was conducted to quantify and characterize the ILC populations in lymphocytes obtained from tumor tissues and peripheral blood. Flow cytometry staining was performed in PBS with 0.5% FBS at 4 °C. Fc receptors were inhibited by incubating cells with Human TruStain FcX™ (BioLegend, Cat# 422302) for a duration of 15 min. Cells were incubated in the dark at 4 °C for 30 min with the following antibodies: FITC anti-human Lineage Cocktail (BioLegend #348801), PE anti-human CD161 Antibody (BioLegend, W18070C #307503), Alexa Fluor® 700 anti-human CD3 Antibody (BioLegend, OKT3 #317339), Alexa Fluor® 700 anti-human CD127 (IL-7Rα) Antibody (BioLegend, A019D5 #351343), Brilliant Violet 421™ anti-human CD117 (c-kit) Antibody (BioLegend, 104D2 #313215), and PE/Cy7 anti-human CD294 (CRTH2) Antibody (BioLegend, BM16 #350117) are listed for reference. Additional surface staining was conducted to compare the expression levels of immune checkpoint molecules PD-1, CTLA-4, and KLRG1, utilizing the specified antibodies. PerCP/Cyanine5.5 anti-human CD152 (CTLA-4) antibody (BioLegend, L3D10 # 349927) APC/Cyanine7 anti-human CD279 (PD-1) Antibody (BioLegend, EH12.2H7 # 329921) and Brilliant Violet 785™ anti-mouse/human KLRG1 (MAFA) Antibody (BioLegend, 2F1/KLRG1 # 138429) are both products from BioLegend. Subsequently, cells underwent two washes with staining buffer. Following centrifugation at 400 × g for 3 min, the supernatant was removed, and the samples were analyzed using a FACS Aria III (BD) flow cytometer.

Intracellular cytokine staining involved stimulating 2 × 10⁶ cells per well with PMA, ionomycin, and Golgi Plug for a duration of 6h. After stimulation, cells were centrifuged at 1500 rpm for 3 min, and the supernatant was discarded. Fixation was conducted with 100 µL of Fixation/Permeabilization Buffer (BD, Cat# 554714) and incubated at 4 °C in the dark for 15 min. Subsequently, 25 µL of permeabilization buffer was added to each well, accompanied by the intracellular antibody APC/Cyanine7 anti-human IFNγ (BioLegend, B27#506523). PE anti-human TNFα (BioLegend, MAb11#986802); PerCP/Cyanine5.5 anti-human IL-2 (BioLegend, MQ1-17H12#500322); PB anti-human GM-CSF (BVD2-21C11#502314); APC anti-human IL-17 (BioLegend, QA18A46#385904). Stained cells were incubated at 4 °C for 30 min in the absence of light. Finally, samples were resuspended in 200 µL of staining buffer and analyzed on a FACS Aria III flow cytometer [18].

### Cell culture and glioma-conditioned medium (GCM) preparation

U87-MG and IDH1-mutant U87-MG cells were seeded at 2 × 10^3^ cells/well in 96-well plates and incubated overnight. Tonsil-derived ILCs (Lineage⁻ CD161⁺ CD127⁺) were sorted by FACSAria III and co-cultured (1 × 10^4^ cells/well) with glioma cells for 4 days, with or without cytokine supplementation (IL-2 10 U/mL, IL-1β 50 ng/mL, IL-23 50 ng/mL, IL-7 50 ng/mL). For GCM preparation, U87-MG and IDH1-mutant U87-MG cells were cultured for 48 h, after which supernatants were collected, filtered (0.22- µm), and supplemented with 5% FBS and antibiotics. Sorted ILCs were cultured in these GCMs (± cytokines) for 3 days and analyzed for PD-1, CTLA-4, and KLRG1 expression and cytokine production.

### Proliferation assay

Prior to co-culture, ILCs were labeled with 5 µM CFSE for 2 min in the dark, washed twice, and cultured as described above. After 4 days, cell proliferation was evaluated using FACS Aria III [19].

### Measurement of D-2-hydroxyglutarate (D-2HG) levels

Plasma samples from IDH1-mutant (n = 7) and IDH1-wildtype (n = 12) glioma patients and healthy controls (n = 12) were collected. GCM from 4-day glioma cell cultures was also analyzed. D-2HG levels were quantified using a colorimetric enzymatic assay (Abcam, Cat# ab211070) per manufacturer’s instructions. Samples were measured in triplicate, and optical density was recorded at 450 nm.

### Mouse experiment, 2-hydroxyglutarate administration, and ILC analysis

To examine the in vivo effects of 2-hydroxyglutarate (2HG) on ILC subsets, male C57BL/6 mice aged 6–8 weeks were randomly allocated to receive daily intraperitoneal injections of D-2HG, L-2HG (20 mg/mL, 100 µL), or PBS for 7 consecutive days (n = 5 per group). The sample size was established by power analysis (effect size = 1.5, α = 0.05, power = 0.8). On day 8, the mice were euthanized, and the small intestine, colon, spleen, and mesenteric lymph nodes were collected. The spleen and lymph nodes underwent mechanical processing, while intestinal tissues were enzymatically digested with Collagenase D (1 mg/mL) and DNase I (0.1 mg/mL) to separate lamina propria lymphocytes. Single-cell suspensions were subsequently enhanced using Percoll gradient centrifugation.

Surface and intracellular labelling was conducted to identify ILC subgroups. Surface staining was performed utilizing the following antibodies: FITC-conjugated anti-mouse Lineage Cocktail with Isotype Control (BioLegend Cat# 133301), APC/Cyanine7-conjugated anti-mouse CD3 (BioLegend Cat# 100221), PE-conjugated anti-mouse CD45 recombinant antibody (BioLegend Cat# 157603), and PE/Cyanine7-conjugated anti-mouse CD90.2 (Thy1.2) (BioLegend Cat# 140309). Cells were treated in PBS with 0.5% FBS at 4 °C for 30 min. Subsequent to surface staining, cells were fixed and permeabilized with eBioscience Fixation/Permeabilization Buffer, followed by intracellular staining utilizing APC-conjugated anti-RORγt (eBioscience Cat# 17-6981-82) and PerCP/Cyanine5.5-conjugated anti-GATA3 (BioLegend Cat# 653811). Following labelling, cells were rinsed and examined utilizing a FACSAria III flow cytometer (BD Biosciences).

### Ex vivo transcription factor analysis of ILCs

Human tonsil-derived innate lymphoid cells were cultured ex vivo with IDH1-wildtype or IDH1-mutant U87 glioma cell lines. Following co-culture, cells were stained for intracellular expression of lineage-associated transcription factors using the BioLegend True-Nuclear Transcription Factor Staining Kit. Pe-conjugated anti-RORγt (eBioscience Cat# 12-6988-82), PeCy7-conjugated anti-T-bet(BioLegend Cat# 365909), and PerCP/Cyanine5.5-conjugated anti-GATA3 (BioLegend Cat# 653811) were used to assess ILC3-, ILC1-, and ILC2-associated transcriptional programs, respectively.

### CD107α degranulation and tumor apoptosis assay

Human tonsil-derived total ILCs were sorted by flow cytometry and cultured overnight in cytokine-supplemented ILC medium. In parallel, lineage-positive cells (containing CD56⁺ NK-cell populations) were sorted and cultured overnight in IL-15–supplemented medium. Glioma target cells (U87-MG and IDH1-Mut-U87) were seeded in 96-well plates 24 h prior to co-culture.

On the day of the assay, effector cells were added to glioma targets at an effector:target (E:T) ratio of 1:2.5. For ILC assays, 10,000 ILCs were co-cultured with 4,000 glioma cells per well. For lineage-positive/NK-containing assays, [NK/lineage⁺ 10,000] effectors were co-cultured with [4000] targets per well at the same E:T ratio. Co-cultures were incubated for 5h in the presence of PE/Dazzle™ 594 anti-human CD107α (LAMP-1) Antibody (BioLegend Cat. # 328645) antibody, followed by flow cytometric analysis of CD107α surface expression on effector cells.

Glioma cells were gated separately at the end of co-culture and stained with Annexin V and 7-AAD to quantify apoptosis/viability. Annexin V and 7-AAD reagents were obtained from (BioLegend Cat. # 640922). The assay was independently repeated using tonsillar samples from five donors.

### Statistics

Data was analyzed with GraphPad Prism software, version-6 and Flowjo (10.0.6). Normality of the data was assessed via Shapiro–Wilk test. Student’s t-test (for normally distributed) and non-parametric Mann–Whitney (for non-normally distributed data) test were used for pairwise comparisons. For multiple comparisons of normally distributed data, ANOVA with Tukey’s post-hoc test was used. For non-normally distributed data, Kruskal–Wallis test with Dunn’s post-hoc correction was used. P value < 0.05 is accepted as significant. (*p ≤ 0.05, **p ≤ 0.01, ***p ≤ 0.001, ****p ≤ 0.0001).

## Results

### Clinical and demographic features of patients

A total of 36 patients diagnosed with diffuse gliomas who underwent surgical resection at the Department of Neurosurgery, Erciyes University, were included in this study. Patient recruitment and sample collection were initiated following ethics committee approval in January 2019 and continued thereafter. Tumors were classified and graded according to the 2021 World Health Organization (WHO) Classification of Central Nervous System Tumors, integrating both histopathological and molecular parameters. For patients diagnosed prior to the publication of the 2021 WHO Classification, tumor samples were retrospectively reviewed and reclassified according to the 2021 WHO criteria for adult-type diffuse gliomas. The clinical and demographic characteristics of the cohort are presented in Table 1. Based on molecular profiling, patients were stratified into three principal diagnostic categories: astrocytoma, IDH-mutant (grades 2–4, n = 7), astrocytoma, IDH-wildtype (grades 2–3, n = 9), and glioblastoma, IDH-wildtype (grade 4, n = 20). All IDH-mutant tumors identified in this cohort harbored IDH1 mutations, and no IDH2-mutant gliomas were detected. The median age of patients with astrocytoma, IDH-mutant ranged from 40 years (range 40–46) in grade 2 to 52.5 years (range 50–55) in grade 3 and 49.5 years (range 45–54) in grade 4. For astrocytoma, IDH-wildtype, the median age was 26 years (range 26–37) in grade 2 and 49 years (range 36–54) in grade 3. The glioblastoma, IDH-wildtype group demonstrated a median age of 49 years (range 28–72).

**Table 1:** Clinical and demographic features of glioma patients

Variable	Astrocytoma, IDH1-mutant grade 2 (n = 3)	Astrocytoma, IDH1-mutant grade 3 (n = 2)	Astrocytoma, IDH1-mutant grade 4 (n = 2)	Astrocytoma, IDH1-wildtype grade 2 (n = 5)	Astrocytoma, IDH1-wildtype grade 3 (n = 4)	Glioblastoma, IDH1-wildtype grade 4 (n = 20)
Age (years)	40 (40–46)	52.5 (50–55)	49.5 (45–54)	26 (26–37)	49 (36–54)	49 (28–72)
Sex (F/M)	2/1	2/0	1/1	1/4	3/1	11/9
Tumor location						
Frontal	0	1	0	0	0	2
Temporal	2	1	1	2	2	7
Parietal/occipital	1	0	1	3	0	10
Other/unspecified	0	0	0	0	2	1
Ki-67 index						
< 10%	1	0	0	4	0	2
≥ 10%	2	2	2	1	4	18

Table 1. Clinical and demographic characteristics of glioma patients according to the 2021 WHO classification. Tumor grading was defined as astrocytoma, IDH1-mutant (grade 2–4) or astrocytoma/glioblastoma, IDH1-wildtype (grade 2–4)

Sex distribution across diagnostic groups was as follows: grade 2 astrocytoma, IDH-mutant (2 F/1 M), grade 3 (2 F/0 M), grade 4 (1 F/1 M); grade 2 astrocytoma, IDH-wildtype (1 F/4 M), grade 3 (3 F/1 M); and glioblastoma, IDH-wildtype (11 F/9 M).

Tumor localization varied across groups. Astrocytoma, IDH-mutant cases were predominantly observed in the temporal (n = 4) and parietal/occipital (n = 2) regions, with one case in the frontal lobe. Astrocytoma, IDH-wildtype lesions were distributed across temporal (n = 4), parietal/occipital (n = 3), and unspecified (n = 2) locations. Among glioblastoma, IDH-wildtype patients, tumors were most frequently found in the parietal/occipital (n = 10) and temporal (n = 7) lobes, followed by the frontal (n = 2) and unspecified (n = 1) regions.

The Ki-67 proliferation index increased consistently with tumor grade. Low proliferative activity (< 10%) was detected in 1/3 of astrocytoma, IDH-mutant grade 2 and 4/5 of astrocytoma, IDH-wildtype grade 2 cases, while all higher-grade astrocytomas and glioblastomas exhibited Ki-67 ≥ 10%.

## IDH1-mutant glioma tissue exhibits reduced ILC1 and enriched ILC3 frequencies, with subset-specific differences in immune checkpoint expression

Innate lymphoid cells (ILCs) are a family of tissue-resident lymphocytes that parallel helper T cell subsets in function but lack antigen receptors. In both humans and mice, ILCs are primarily located at barrier surfaces such as the gut, skin, and lungs, yet they are also found in lymphoid and non-lymphoid tissues, including the central nervous system [20].

To examine ILC subset distribution in gliomas, we analyzed mononuclear cells isolated from resected tumor tissues of patients classified according to the 2021 WHO CNS tumor classification (grade II–IV gliomas) and compared them to tonsillar tissues obtained from individuals undergoing routine tonsillectomy for recurrent tonsillitis. All tumor and tonsil samples were cryopreserved prior to staining and thawed on the day of flow cytometric analysis. Samples yielding < 1 × 10⁶ viable lymphocytes post-thaw were excluded from analysis to ensure data reliability.

**Fig. 1:**
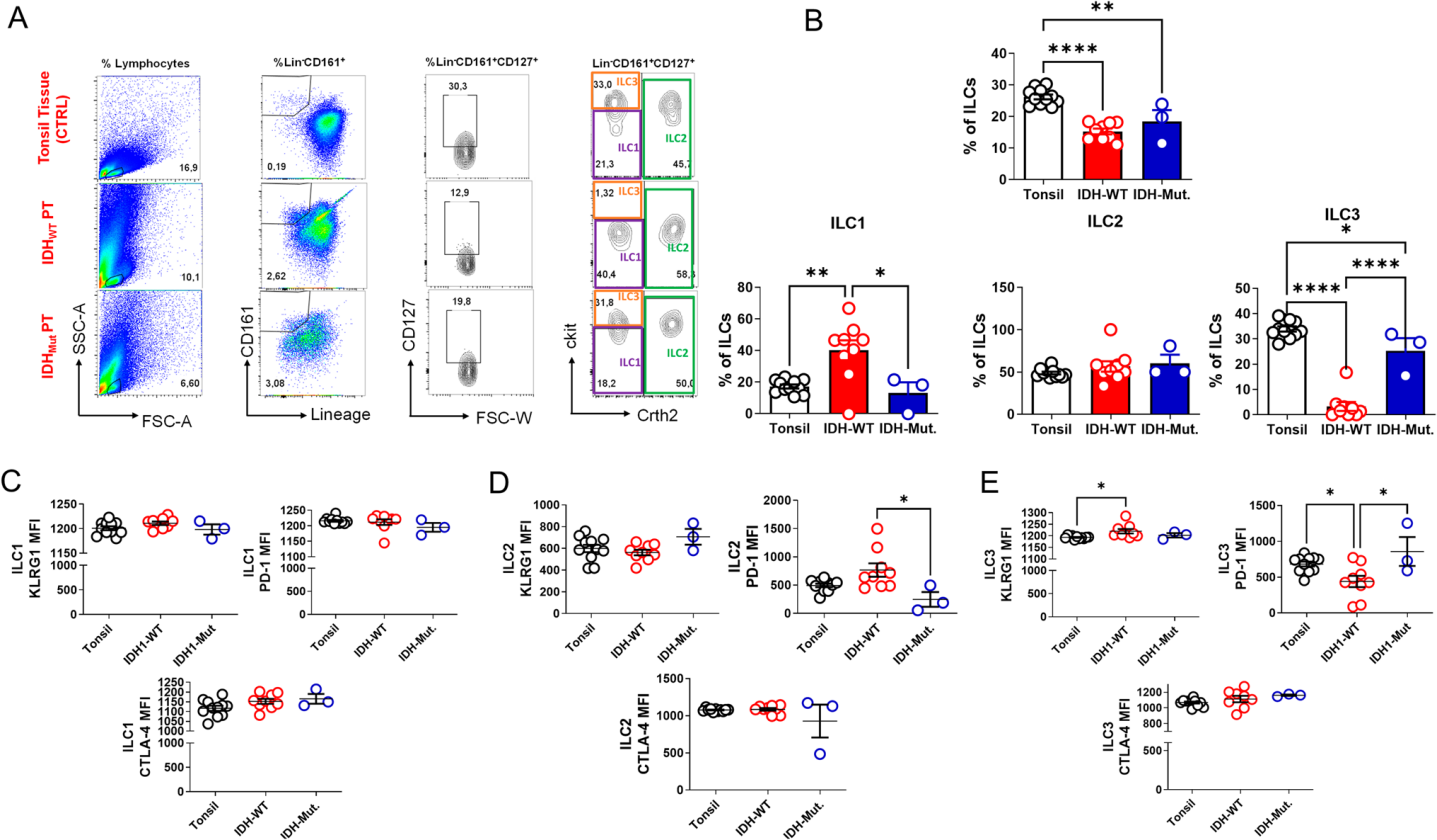
The frequency of ILC1 is decreased, whereas ILC3 is increased, in IDH1-mutant human glioma tissues compared with the IDH1-wild-type group. **A** Representative flow-cytometry gating strategies for ILCs in human tonsil (control) tissue, IDH1-wildtype, and IDH1-mutant glioma tissue (FSC-A = forward-scatter area; SSC-A = side-scatter area). **B** Comparison of total ILC percentages in tonsil controls (n = 11), IDH1-wild-type (n = 9), and IDH1-mutant (n = 3) glioma tissues (top center); comparison of ILC1 percentages (bottom left), ILC2 percentages (bottom center), and ILC3 percentages (bottom right) across the same groups. **C** Comparison of mean fluorescence intensity (MFI) of KLRG1 (top left), PD-1 (top right), and CTLA-4 (bottom center) expression on ILC1s in tonsil control, IDH1-wild-type, and IDH1-mutant groups. **D** Comparison of MFI of KLRG1 (top left), PD-1 (top right), and CTLA-4 (bottom center) on ILC2s. **E** Comparison of MFI of KLRG1 (top left), PD-1 (top right), and CTLA-4 (bottom center) on ILC3s. Error bars represent ± SD. Statistical significance was determined by one-way ANOVA (*p ≤ 0.05, **p ≤ 0.01, ***p ≤ 0.001, ****p ≤ 0.0001)

ILCs were defined as live Lin⁻CD161⁺CD127⁺ lymphocytes, and subsets were identified based on CRTH2 and c-Kit expression: ILC1 (CRTH2⁻c-Kit⁻), ILC2 (CRTH2⁺), and ILC3 (CRTH2⁻c-Kit⁺). Representative gating hierarchy is shown in Fig. 1A. All forward and side scatter plots were displayed on linear scales for consistency. Gating thresholds were defined based on unstained and isotype control samples processed in parallel, as previously described in similar tissue-based ILC analyses.

Frequencies of total and subset ILCs were calculated as a percentage of total viable lymphocytes. The overall proportion of total ILCs was significantly lower in glioma tissues than in tonsillar controls, with the most pronounced reduction observed in IDH1-wildtype tumors. Among subsets, ILC1 frequencies were markedly decreased in IDH1-mutant gliomas compared with IDH1-wildtype gliomas. In contrast, ILC3 frequencies were significantly elevated in IDH1-mutant gliomas relative to IDH1-wildtype gliomas, whereas ILC2 frequencies remained comparable across all groups (Fig. 1B).

Expression of immune checkpoint receptors—KLRG1, PD-1, and CTLA-4—was also analyzed on each ILC subset. ILC1s displayed similar levels of all three checkpoint molecules across IDH1-mutant, IDH1-wildtype, and tonsil control groups (Fig. 1C). ILC2s from IDH1-mutant gliomas exhibited significantly reduced PD-1 expression compared to IDH1-wildtype gliomas (Fig. 1D). For ILC3s, KLRG1 expression was elevated in IDH1-wildtype gliomas compared to tonsil controls, while PD-1 expression was higher in IDH1-mutant gliomas than in IDH1-wildtype tumors (Fig. 1E). CTLA-4 expression did not differ significantly among groups. Together, these findings indicate that IDH1-mutant gliomas harbor a distinct ILC landscape characterized by reduced total ILC and ILC1 populations, enrichment of ILC3s, and subset-specific alterations in checkpoint molecule expression.

### Peripheral blood ILC3 frequency is elevated, and immune checkpoints are differentially expressed in IDH1-mutant glioma patients

**Fig. 2:**
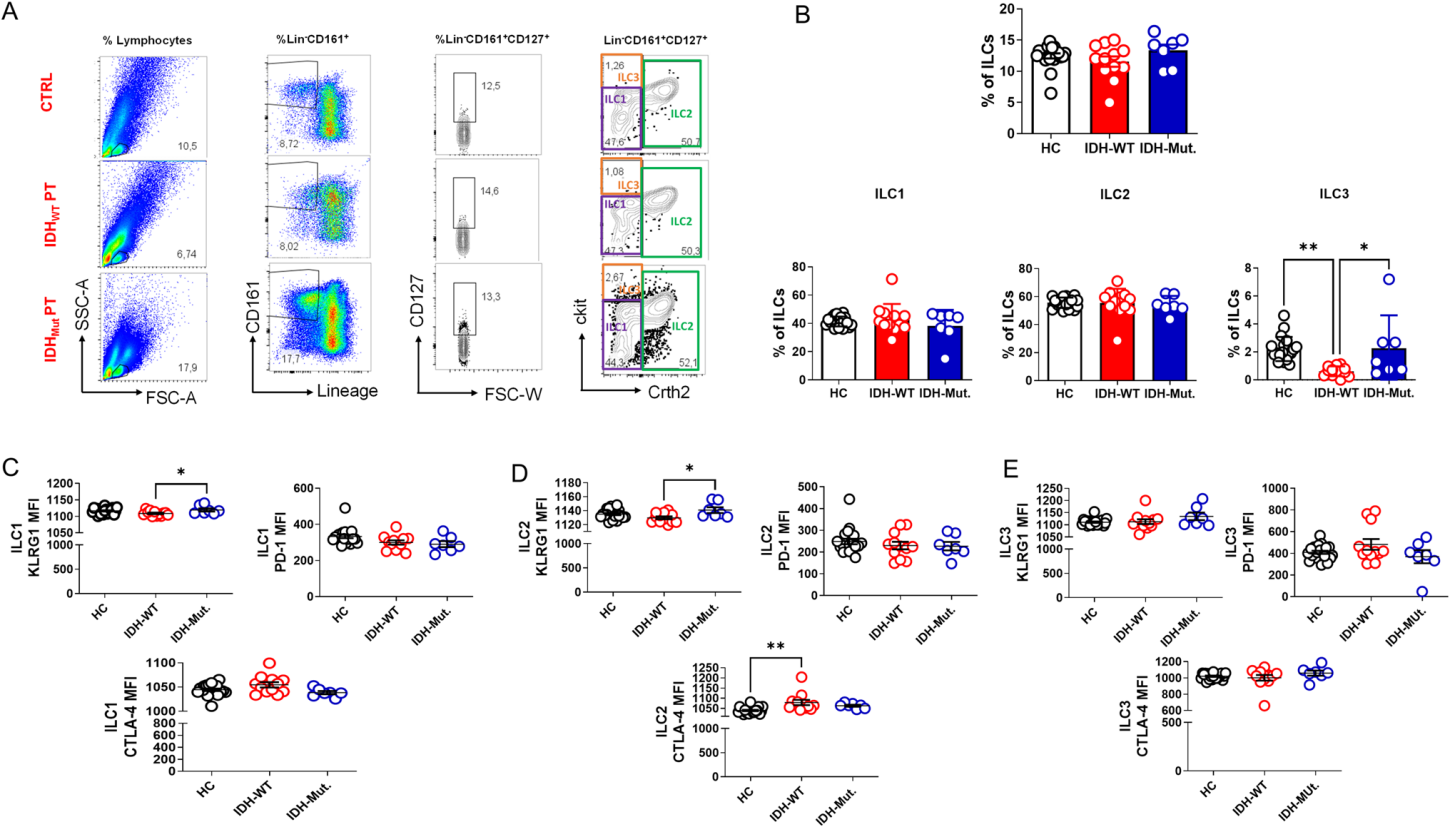
The frequency of ILC3 is increased in the peripheral blood of IDH1-mutant glioma patients compared with the IDH1-wild-type group. **A** Representative flow-cytometry gating strategies for ILCs in peripheral blood of healthy controls, IDH1-wild-type glioma patients, and IDH1-mutant glioma patients (FSC-A = forward-scatter area; SSC-A = side-scatter area). **B** Comparison of total ILC percentages in blood samples from healthy controls (HC, n = 20), IDH1-wild-type (n = 12), and IDH1-mutant (n = 7) groups (top center); comparison of ILC1 (bottom left), ILC2 (bottom center), and ILC3 (bottom right) frequencies across the same groups. **C** Comparison of MFI of KLRG1 (top left), PD-1 (top right), and CTLA-4 (bottom center) on ILC1s in HC, IDH1-wild-type, and IDH1-mutant groups. **D** Comparison of MFI of KLRG1 (top left), PD-1 (top right), and CTLA-4 (bottom center) on ILC2s. **E** Comparison of MFI of KLRG1 (top left), PD-1 (top right), and CTLA-4 (bottom center) on ILC3s. Error bars represent ± SD. Statistical significance was determined by one-way ANOVA (*p ≤ 0.05, **p ≤ 0.01, ***p ≤ 0.001, ****p ≤ 0.0001)

Peripheral blood ILCs from patient and healthy control groups were also analyzed with respect to number, frequency, and immune checkpoint receptor expression. The flow cytometry gating strategy for peripheral blood ILCs is given in (Fig. 2A). There was a significantly higher frequency of ILC3 in the blood of IDH1-mutant glioma patients compared to IDH1-wildtype glioma patients, consistent with the tumor microenvironment. Simultaneously, a reduced ILC3 population was observed in the blood of patients with IDH1-wildtype glioma compared to healthy controls. Total ILC, ILC1, and ILC2 percentages were similar in all three groups (Fig. 2B).

Analyses of KLRG1, PD1, and CTLA4 expression in peripheral blood ILC subgroups showed increased KLRG1 expression by ILC1 and ILC2 in IDH1-mutant patients compared to the IDH1-wildtype group (Fig. 2C, D). IDH1-wildtype ILC2 CTLA4 expression was increased compared to healthy controls (Fig. 2D). However, KLRG1, PD1, and CTLA4 expressions in ILC3 were similar in all three groups (Fig. 2E).

Altogether, these data are in line with the tissue ILC profile, supporting a bias toward ILC3 in IDH1-mutant glioma patients and differentially upregulated expression of immune checkpoints by distinct ILC subsets.

### Co-culture of ILCs with IDH1-mutant or wild-type glioma cells and exposure to their glioma-conditioned medium (GCM) alter immune checkpoint expression and proliferation of ILCs

**Fig. 3:**
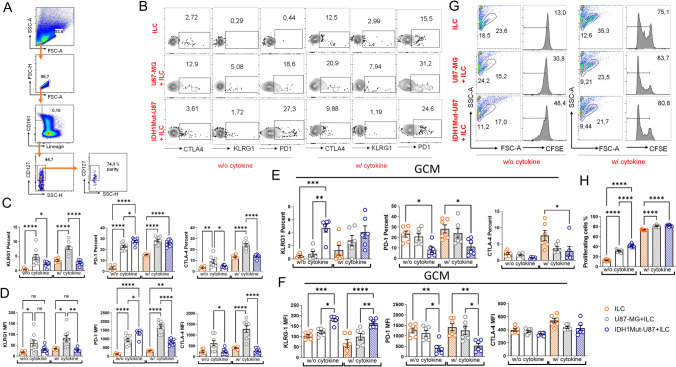
Co-culture of tonsil-derived ILCs with U87-MG significantly increased surface PD-1, KLRG1, and CTLA-4 compared with IDH1-mutant U87-MG. **A**–**D** Human tonsil-derived ILCs were cultured alone or co-cultured with U87-MG or IDH1-mutant U87-MG glioma cell lines, either with cytokine supplementation (recombinant human IL-2 [5 ng/mL], IL-7 [50 ng/mL], IL-12 [50 ng/mL], IL-1β [50 ng/mL], IL-23 [50 ng/mL]) or without cytokines for four days. Data points represent two independent experiments (ILC n = 3; U87-MG + ILC n = 6; IDH1-mutant U87-MG + ILC n = 6; technical replicates) **A** Representative flow-cytometry gating strategy for human tonsil-derived ILCs.** B** Flow-cytometry contour plots showing CTLA-4, KLRG1, and PD-1 surface expression percentages on ILCs. **C** Quantification of CTLA-4, KLRG1, and PD-1 expression percentages on ILCs. **D** Mean fluorescence intensity (MFI) of CTLA-4, KLRG1, and PD-1 expression on ILCs. **E**–**F** ILCs were cultured alone or exposed to glioma-conditioned medium (GCM) from U87-MG or IDH1-mutant U87-MG cell lines under the same cytokine conditions for four days. Data points represent two independent experiments (each with three technical replicates). **E** Percentages of CTLA-4, KLRG1, and PD-1–expressing ILCs following GCM exposure. **F** Corresponding MFI of CTLA-4, KLRG1, and PD-1 expression on ILCs. **G**–**H** Proliferation of CFSE-labeled tonsil ILCs co-cultured with U87-MG or IDH1-mutant U87-MG cells was analyzed after four days. **G** Representative flow-cytometry plots of CFSE dilution. **H** Quantification of proliferating CFSE-labeled ILCs from three independent experiments (each with four technical replicates). Error bars represent ± SD. Statistical analyses were performed using one-way ANOVA (*p ≤ 0.05, **p ≤ 0.01, ***p ≤ 0.001, ****p ≤ 0.0001)

To better assess how IDH1-mutant and IDH1-wild-type glioma cells influence innate lymphoid cells (ILCs) outside the tumor microenvironment, we co-cultured U87-MG and isogenic IDH1-mutant U87 cell lines with human tonsil-derived total ILCs for two days. A representative gating strategy for these co-cultures is shown in Fig. 3A. Culture conditions included cytokines that support ILC survival and expansion (IL-2 [10 U/mL], IL-1β [50 ng/mL], IL-23 [50 ng/mL], IL-7 [50 ng/mL]). After three days of co-culture, KLRG1, CTLA-4, and PD-1 expression on ILCs was evaluated by flow cytometry (Fig. 3B). The frequency of KLRG1⁺ and CTLA-4⁺ ILCs decreased in co-cultures with IDH1-mutant U87 compared to U87-MG under both cytokine-supplemented and cytokine-free conditions (Fig. 3C). Similarly, the mean fluorescence intensity (MFI) of KLRG1, CTLA-4, and PD-1 was reduced in cultures with IDH1-mutant U87 relative to U87-MG (Fig. 3D). In contrast, ILCs co-cultured with U87-MG showed higher checkpoint expression than ILCs cultured alone (Fig. 3C, D).

To test whether direct cell-to-cell contact was required, glioma-conditioned media (GCM) were collected from U87-MG and IDH1-mutant U87 cultures without disturbing the adherent monolayer. To preserve viability and prevent contamination, the GCM was supplemented with 5% FBS and 1 × penicillin–streptomycin and filtered through a 0.2 µm membrane. ILCs were then incubated with GCM from both glioma cell lines for three days. Unlike direct co-cultures, both the frequency and MFI of KLRG1⁺ ILCs increased when exposed to IDH1-mutant U87 GCM compared with U87-MG GCM. Similar to the cellular co-culture experiments, PD-1 and CTLA-4 expression tended to decrease in the presence of IDH1-mutant U87 GCM (Fig. 3E, F).

In additional assays, ILC proliferation was quantified after four days of co-culture using CFSE dilution. Proliferation of ILCs was significantly higher when cultured with IDH1-mutant U87 cells compared with U87-MG, consistent with lower immune checkpoint receptor expression (Fig. 3H). Collectively, these findings indicate that the IDH1-mutant glioma microenvironment exhibits a reduced capacity to upregulate immune checkpoint receptors on ILCs while promoting their proliferative activity ex vivo.

To determine whether altered checkpoint expression translated into functional consequences, we performed a CD107α-based cytotoxicity assay following short-term co-culture with glioma cells. Sorted tonsil-derived ILCs were co-cultured with glioma targets at an effector:target ratio of 1:2.5 for 5h (Supplementary Fig. 6A–G).

ILCs exhibited measurable CD107α upregulation upon exposure to glioma cells. Unexpectedly, ILCs and NK cells showed reduced CD107α degranulation in IDH1-mutant U87 cultures (Supplementary Fig. 6B, C). Analysis of target cells at the end of co-culture revealed significantly reduced apoptosis in the IDH1-mutant condition compared to IDH1-wildtype co-cultures, as assessed by Annexin V/7-AAD staining (Supplementary Fig. 6D, G).

These findings demonstrate that although reduced checkpoint inhibitor expression in IDH1-mutant glioma-ILC cocultures and 2-HG conditioned media correlates with higher ILC proliferation, this does not translate to more potent cytotoxic activity for ILCs as a functional outcome.

### Glioma-conditioned medium (GCM) enhances cytokine production by ILCs regardless of IDH1 mutation status.

To investigate whether soluble factors released from IDH1-mutant and IDH1-wild-type glioma cells differentially influence human ILC effector responses, tonsil-derived ILCs were cultured with glioma-conditioned medium (GCM) collected from IDH1-mutant U87-MG and U87-MG cell lines. Intracellular cytokine production was quantified by flow cytometry.

**Fig. 4:**
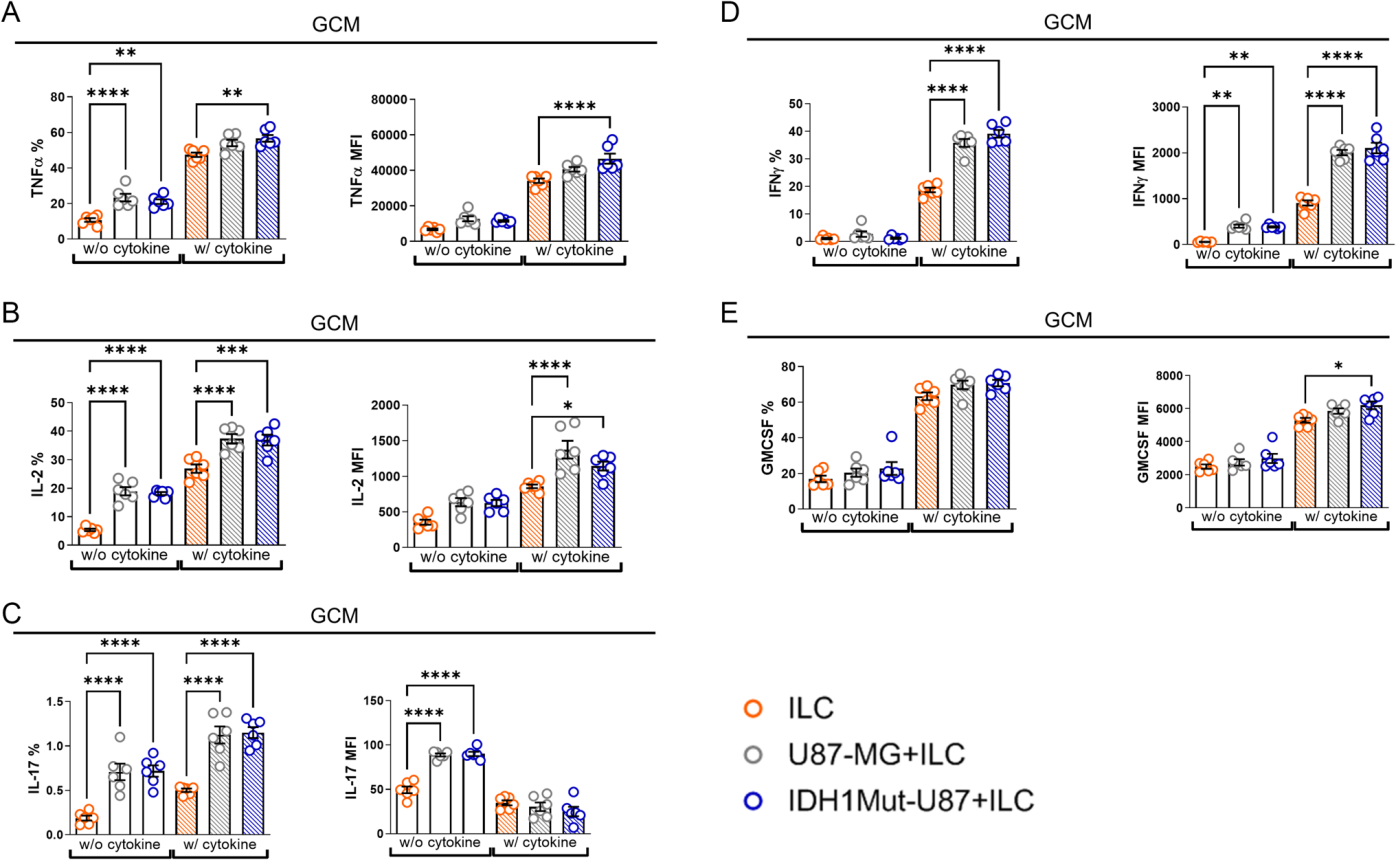
IL-17 and IFN-γ production is increased in tonsil-derived ILCs exposed to glioma-conditioned medium (GCM) from U87-MG and IDH1-mutant U87-MG cell lines. **A**–**E** Tonsil-derived ILCs were cultured alone or with glioma-conditioned medium (GCM) obtained from U87-MG or IDH1-mutant U87-MG cell lines for four days, with or without cytokine supplementation (recombinant human IL-2 [5 ng/mL], IL-7 [50 ng/mL], IL-12 [50 ng/mL], IL-1β [50 ng/mL], IL-23 [50 ng/mL]). Golgi Stop was added during the final nine hours of incubation. **A** Percentages (left) and mean fluorescence intensity (MFI; right) of TNF-α–producing ILCs. **B** Percentages (left) and MFI (right) of IL-2–producing ILCs. **C** Percentages (left) and MFI (right) of IL-17–producing ILCs. **D** Percentages (left) and MFI (right) of IFN-γ–producing ILCs. **E** Percentages (left) and MFI (right) of GM-CSF–producing ILCs. Data represents two independent experiments (each with three technical replicates). Error bars indicate ± SEM. Statistical significance was determined by one-way ANOVA (*p ≤ 0.05, **p ≤ 0.01, ***p ≤ 0.001, ****p ≤ 0.0001)

ILCs exposed to either IDH1-mutant U87-MG GCM or U87-MG GCM showed a marked increase in the production of TNF-α, IL-2, IL-17, and IFN-γ compared with ILCs cultured in standard medium (Fig. 4A–E, Supplementary Fig. 1). Supplementation with ILC-supporting cytokines further amplified these responses, particularly for IL-17 and IFN-γ, both associated with type 3 ILC polarization.

In contrast, GM-CSF production remained relatively consistent across all culture conditions (Fig. 4E). Although minor increases were observed upon cytokine supplementation, exposure to either form of GCM did not significantly alter its expression levels.

Collectively, these findings suggest that soluble glioma-derived factors promote a general activation of ILC effector functions, independent of the IDH1 mutation status. Despite the metabolic differences between mutant and wild-type glioma cells, ILCs appear to preserve their intrinsic cytokine-producing potential within tumor-derived soluble environments.

To further address the potential impact of ILC plasticity and to validate lineage identity beyond surface markers, we performed transcription factor–based analysis following ex vivo co-culture with glioma cells. Sorted human tonsil-derived ILCs were cultured with IDH1-wildtype or IDH1-mutant U87 cells for 3 days and subsequently analyzed for intracellular expression of lineage-associated transcription factors (Supplementary Fig. 5).

As shown in Supplementary Fig. 5B–C, co-culture with IDH1-mutant glioma cells resulted in a significant reduction in T-bet–defined ILC1s and a relative enrichment of both RORγt-expressing ILC3s and ILC2-associated transcriptional populations compared to the IDH1-wildtype condition. These findings support that the phenotypic shifts observed in the main figures are accompanied by corresponding lineage-associated transcriptional programs rather than reflecting transient modulation of surface markers alone.

### D-2-HG levels in IDH1-mutant and IDH1-wildtype glioma patient plasma and glioma-conditioned media

**Fig. 5:**
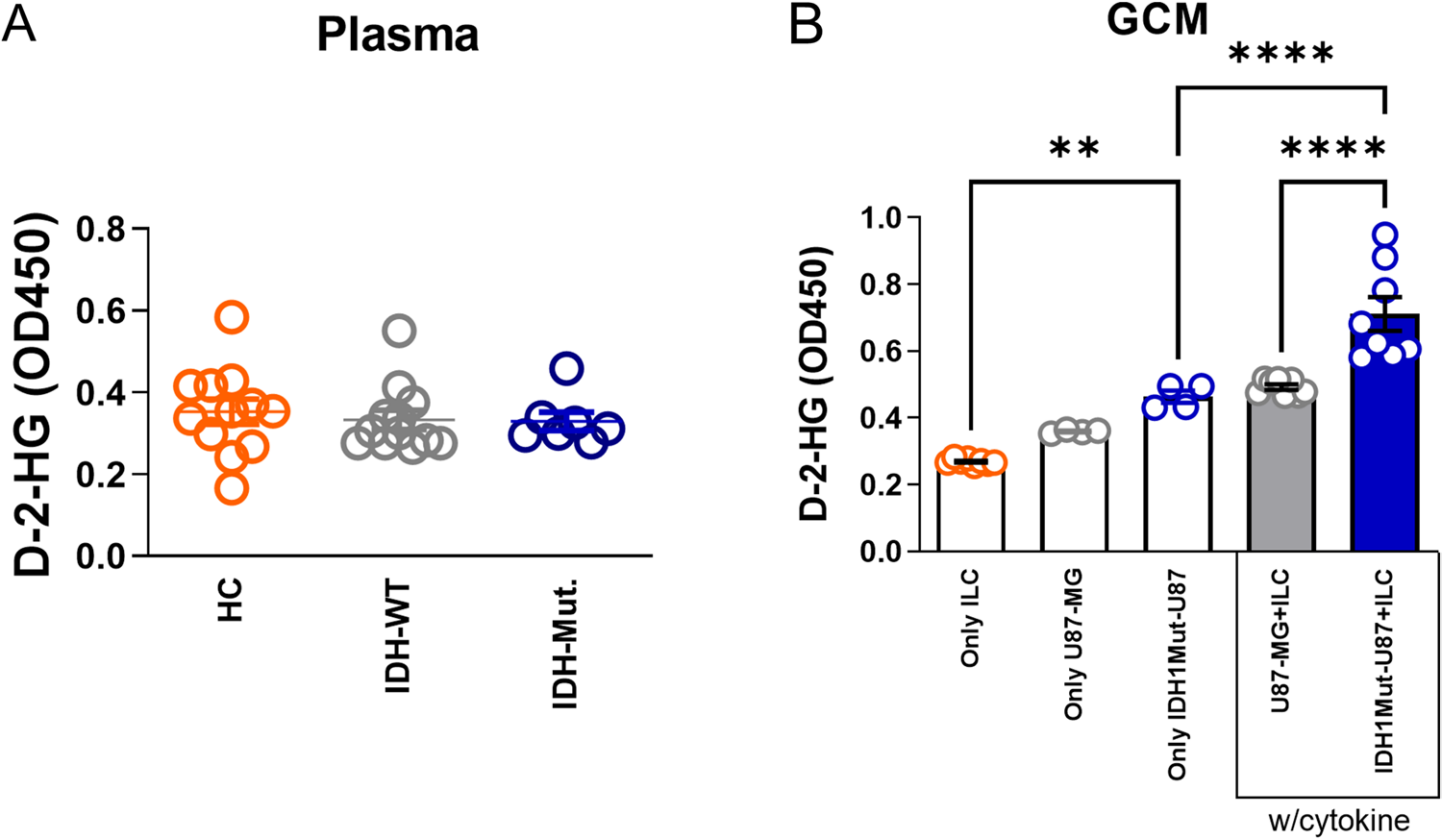
D-2-HG levels are comparable in-patient plasma but elevated in glioma-conditioned medium (GCM) from IDH1-mutant U87-MG + ILC co-cultures compared with U87-MG + ILC. **A** Quantification of D-2-HG (OD₄₅₀) in plasma samples from healthy controls (HC, n = 12), IDH1-wild-type glioma patients (n = 12), and IDH1-mutant glioma patients (n = 7). **B** Quantification of D-2-HG (OD₄₅₀) in glioma-conditioned medium (GCM) collected from cultures of ILCs alone, U87-MG, IDH1-mutant U87-MG cell lines, and tonsil ILCs co-cultured with U87-MG or IDH1-mutant U87-MG cells under cytokine-supplemented (recombinant human IL-2 [5 ng/mL], IL-7 [50 ng/mL], IL-12 [50 ng/mL], IL-1β [50 ng/mL], IL-23 [50 ng/mL]) and cytokine-free conditions for four days. Data points represent two independent experiments (ILC n = 4, U87-MG + ILC n = 2, IDH1-mutant U87-MG + ILC n = 2 technical replicates). Error bars indicate ± SEM. (*p ≤ 0.05, **p ≤ 0.01, ***p ≤ 0.001, ****p ≤ 0.0001)

To assess the relationship between IDH1 mutation status and D-2-hydroxyglutarate (D-2-HG) accumulation, plasma samples were analyzed from patients with IDH1-mutant and IDH1-wildtype glioma as well as from healthy controls. D-2-HG optical density (OD₄₅₀) values did not differ significantly among the three groups (Fig. 5A). In contrast, D-2-HG levels were significantly higher in GCM from IDH1-mutant U87-MG + ILC co-cultures than in either IDH1-mutant U87-MG alone or U87-MG + ILC groups (Fig. 5B). These data indicate that D-2-HG accumulation occurs primarily within the local glioma microenvironment rather than systemically in the peripheral circulation.

### D-2-HG exposure alters immune checkpoint expression, proliferation, and cytokine production in human ILCs

To determine whether D-2-hydroxyglutarate (D-2-HG) directly influences ILC phenotype, tonsil-derived ILCs were cultured ex vivo for five days in increasing concentrations of D-2-HG (2, 4, and 6 mg/mL). At the highest concentration (6 mg/mL), D-2-HG induced noticeable apoptosis (Supplementary Fig. 2).

**Fig. 6:**
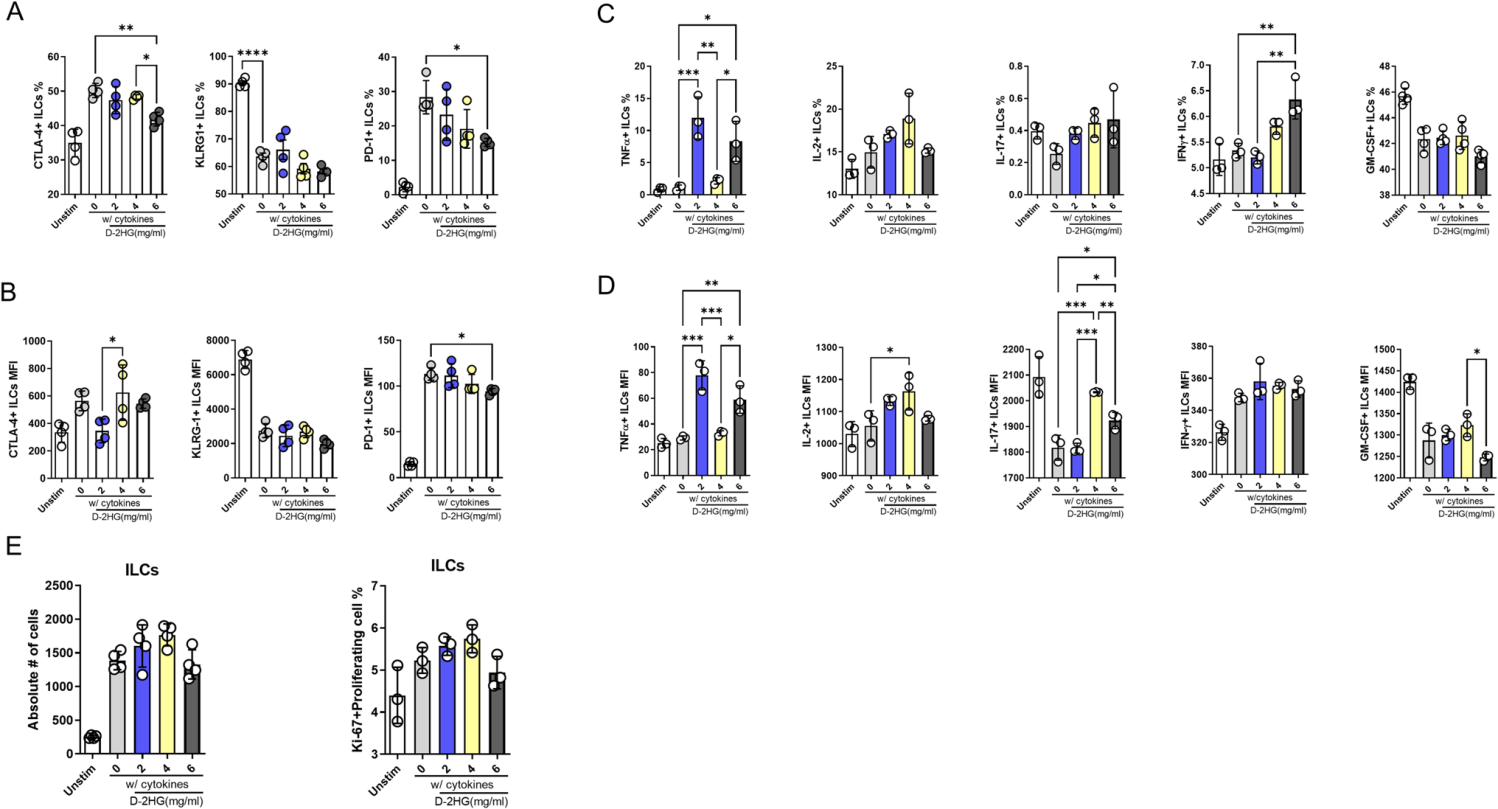
Exposure of tonsil-derived ILCs to D-2-HG decreases CTLA-4 and PD-1 expression and differentially modulates cytokine production. **A**–**E** Human tonsil-derived ILCs were cultured with increasing concentrations of D-2-HG (0, 2, 4, 6 mg/mL) for four days, with or without cytokine supplementation (recombinant human IL-2 [5 ng/mL], IL-7 [50 ng/mL], IL-12 [50 ng/mL], IL-1β [50 ng/mL], IL-23 [50 ng/mL]). **A** Percentages of CTLA-4⁺, PD-1⁺, and KLRG1⁺ ILCs. **B** Mean fluorescence intensity (MFI) of CTLA-4, PD-1, and KLRG1 on ILCs. **C** Percentages of TNF-α-, IL-2-, IL-17A-, IFN-γ-, and GM-CSF-producing ILCs. **D** MFI values for the same cytokines. **E** Absolute cell numbers and Ki-67⁺ ILC percentages at indicated D-2-HG concentrations. Data represent a single experiment performed in technical triplicate. Error bars indicate ± SEM. (*p ≤ 0.05, **p ≤ 0.01, ***p ≤ 0.001, ****p ≤ 0.0001; one-way ANOVA)

Flow cytometric analysis showed a dose-dependent reduction in CTLA-4 and PD-1 expression on ILCs, while KLRG1 expression remained relatively unchanged (Fig. 6A, B). Exposure to higher D-2-HG concentrations also led to a moderate increase in IFN-γ and IL-2 production, whereas IL-17A and GM-CSF levels were not significantly altered (Fig. 6C, D).

Analysis of proliferation revealed a slight, non-significant reduction in total cell number and Ki-67⁺ proliferating ILCs at 6 mg/mL D-2-HG (Fig. 6E, Supplementary Fig. 3).

Overall, these data indicate that D-2-HG selectively downregulates checkpoint receptor expression while modestly enhancing certain cytokine responses, suggesting that additional soluble mediators present in the IDH1-mutant glioma microenvironment may contribute to the enhanced ILC proliferation observed in co-culture experiments.

### 2-HG administration alters the distribution of ILC subsets in vivo

**Fig. 7:**
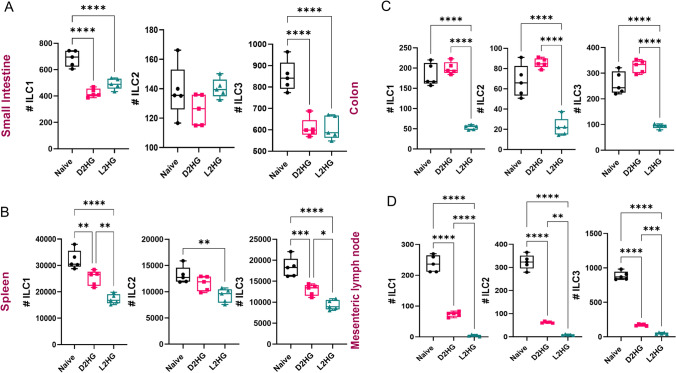
D-2-HG and L-2-HG administration alters ILC subset abundance across murine tissues. **A**–**D** Absolute numbers of ILC1, ILC2, and ILC3 were quantified in different organs of naïve, D-2-HG-treated, and L-2-HG-treated C57BL/6 mice. **A** Small intestine (SI). **B** Spleen. **C** Colon. **D** Mesenteric lymph nodes (MLN). Data are shown as box plots with individual points representing single animals (n = 5 per group). Boxes indicate the interquartile range with median lines; whiskers show minimum–maximum values. Statistical significance was determined by one-way ANOVA (*p ≤ 0.05, **p ≤ 0.01, ***p ≤ 0.001, ****p ≤ 0.0001)

To investigate the in vivo impact of 2-hydroxyglutarate (2-HG) on innate lymphoid cells (ILCs), naïve C57BL/6 mice were intraperitoneally injected with either D-2-HG or L-2-HG for seven consecutive days (Supplementary Fig. 4A). Absolute ILC subset counts were quantified in the small intestine (SI), spleen, colon, and mesenteric lymph nodes (MLNs). In the small intestine, both D-2-HG and L-2-HG markedly reduced the numbers of ILC1 and ILC3, whereas ILC2 numbers remained unchanged (Fig. 7A). In the spleen, both enantiomers significantly decreased ILC1 and ILC3 numbers, while ILC2 reduction was observed only after L-2-HG exposure (Fig. 7B). In the colon, L-2-HG administration significantly decreased all three ILC subsets (ILC1, ILC2, and ILC3) (Fig. 7C). In the MLNs, both D-2-HG and L-2-HG significantly reduced ILC1, ILC2, and ILC3 counts (Fig. 7D). Similar reductions were also observed in relative frequencies (Supplementary Fig. 4). Although this in vivo system does not recapitulate glioma, and glioma-driven systemic and local tumor microenvironment, as a proof of principle, these data demonstrate that both stereoisomers of 2-HG exert a negative effect on ILC abundance across mucosal and secondary lymphoid tissues in vivo.

## Discussion

In the current study, we investigated for the first time the frequency of ILCs and their expression of the checkpoint molecules KLRG1, PD-1, and CTLA-4 in human patients’ glial tumors and peripheral blood, comparing IDH1 wildtype (WT) and mutant (Mut) gliomas. To further delineate the effect of the IDH1 mutation and its metabolite D-2HG on ILCs, we performed complementary in vitro co-culture experiments and in vivo exposure models, demonstrating that IDH-associated oncometabolites influence ILC populations across tumor and peripheral tissues. The tumor microenvironment in gliomas is known to exhibit significant immune exhaustion, especially among T cells, which show diminished function and poor proliferation [21]. While previous studies have focused mainly on the adaptive immune compartment, our work extends this concept to innate lymphoid cells (ILCs), uncovering how their frequency, checkpoint receptor expression, and cytokine function differ between IDH1-mutant and wild-type gliomas.

Group 1 ILCs, which include cytotoxic NK cells and helper ILC1s, are key players in anti-tumor immunity. NK cells mediate direct cytotoxicity against tumor cells [22] while ILC1s, although less cytotoxic, contribute through cytokine production, including IFN-γ and TNF-α, and by recruiting other immune cells [23, 24]. ILC1s have been implicated in both tumor suppression [25, 26] and promotion [27, 28] depending on the context. In hematological malignancies such as AML and CML, increased ILC1 or ILC1-like populations have been documented [29, 30]. Our study shows that in IDH1 Mut gliomas, the frequency of ILC1s was significantly reduced in the tumor tissue compared to both IDH1 WT gliomas and tonsillar controls, while peripheral blood ILC1 frequencies remained comparable to healthy controls. These findings suggest that IDH1 mutations create a local, but not systemic, suppressive niche affecting ILC1s.

ILC2s are typically activated by epithelial-derived cytokines such as IL-25, IL-33, and TSLP, [31] and can interact with tumor cells via NKp30 [31]. Although predominantly known for their roles in allergy and helminth defense, ILC2s can exert both pro-tumor [32–42] and anti-tumor [43–49] effects. In our study, ILC2 frequencies remained unchanged across all patient groups, suggesting that this subset may be less responsive to the IDH1 mutation or its associated environmental cues.

ILC3s, characterized by NKp44 (human) or NKp46 (mouse), exhibit a dual role in tumor biology. Pro-tumor functions of ILC3s have been associated with IL-22-mediated support of tumor growth, epithelial proliferation, and chronic inflammation in models of colorectal, breast, hepatic, and cervical cancers [50–56]. Conversely, anti-tumor activities include antigen presentation via MHC-II, promotion of tertiary lymphoid structures, and support of TH1 immunity [57–59]. In agreement with these reports, our data demonstrate a robust enrichment of ILC3s in both the tumor tissue and peripheral blood of IDH1-mutant glioma patients compared to IDH1-wildtype and tonsil controls. This systemic elevation of ILC3s may reflect enhanced migration from the tumor site or systemic imprinting by IDH1-mutant gliomas.

Considering the shift from ILC1 to ILC3 dominance in IDH1-mutant gliomas, cytokines such as TGF-β and IL-23 may drive plasticity between these subsets [60, 61]. However, because NK cells were excluded from our gating strategy, future research should evaluate NK–ILC1–ILC3 transdifferentiation and the cytokines present in the local milieu that regulate this plasticity.

Checkpoint receptor expression modulates immune cell activity. PD-1, CTLA-4, and KLRG1 are all known to inhibit cytokine production and proliferation of T cells and ILCs [62]. Previous reports have shown elevated PD-1 and CTLA-4 expression in tumor-infiltrating ILC2s [63] and ILC3s [64] as well as increased KLRG1 expression in multiple cancers [65]. In our cohort, PD-1 expression was significantly increased in ILC3s but reduced in ILC2s from IDH1-mutant gliomas. Moreover, KLRG1 expression was elevated in circulating ILC1s and ILC2s, suggesting that the IDH1 mutation induces subset-specific checkpoint modulation. These findings align with prior observations describing altered checkpoint expression among ILCs in cancer [66–70].

To evaluate whether glioma cells directly influence ILC phenotype, we cultured human tonsil-derived ILCs with U87 (IDH1 WT) or IDH1-mutant U87 cells. These co-culture systems were used to model the direct cellular interactions between glioma cells and ILCs under controlled in vitro conditions, providing a simplified representation of the tumor–immune interface. Compared with wild-type glioma cells, IDH1-mutant cells induced a consistent downregulation of checkpoint molecules on ILCs. Interestingly, KLRG1 expression increased in response to glioma-conditioned medium (GCM) alone, while PD-1 and CTLA-4 levels decreased, indicating that soluble mediators released by IDH1-mutant glioma cells exert differential effects on checkpoint receptor regulation. These results suggest that the IDH1-mutant glioma microenvironment selectively reprograms ILCs through distinct soluble and metabolic factors rather than causing uniform immunosuppression.

The differential behavior of checkpoint receptors observed in direct co-culture versus conditioned-media experiments highlights the mechanistic complexity of glioma–ILC interactions. KLRG1 is a well-characterized inhibitory receptor expressed on NK cells and subsets of ILCs that binds classical cadherins, including E-cadherin, N-cadherin, and R-cadherin, on target cells. Engagement of KLRG1 by these membrane-bound ligands requires cell–cell contact and transduces inhibitory signals that attenuate effector responses [71].

In addition to juxtacrine signaling, soluble forms of E-cadherin have been reported to influence KLRG1-mediated regulation, suggesting that this receptor may be modulated not only by direct cellular contact but also, under certain conditions, by diffusible factors. These properties are consistent with our observation that KLRG1 displayed distinct regulation patterns in direct co-culture compared with glioma-conditioned media [72].

Direct co-culture integrates cell–cell contact, membrane-bound ligand engagement, and localized metabolite gradients, whereas conditioned media primarily capture soluble effects. Accordingly, Fig. 3 supports mechanistic heterogeneity rather than a single dominant pathway regulating ILC checkpoint expression. Importantly, our experimental system does not formally dissect contact-dependent from soluble mechanisms; future studies employing transwell systems or ligand-blocking strategies will be required to define these pathways more precisely [71].

The enhanced proliferation of ILCs co-cultured with IDH1-mutant glioma cells further indicates a distinct immunomodulatory signature. This finding aligns with the clinical observation that IDH1-mutant gliomas exhibit a less immunosuppressive tumor microenvironment and are associated with improved patient outcomes.

D-2HG, a hallmark oncometabolite of IDH1/2 mutations, was originally implicated in gliomagenesis through epigenetic and metabolic reprogramming [73–75]. It has also been shown to affect T cell differentiation, cytokine production, and survival [76–78].

In line with prior literature, our results confirm that D-2HG accumulation is localized within the tumor microenvironment rather than detectable systemically [70, 73]. Elevated D-2HG in IDH1-mutant U87 GCM correlated with increased ILC3 frequency, suggesting a local role for this metabolite in shaping ILC profiles.

Previous studies have reported divergent effects of D-2HG on immune regulation, including reduced T-cell infiltration in IDH1-mutant gliomas and altered Treg/Th17 balance [74–78]. We found that D-2HG exposure downregulated PD-1 and CTLA-4 expression on ILCs while simultaneously increasing IFN-γ and TNF-α production. These findings indicate that, although D-2HG may inhibit T cell responses, it might enhance ILC effector function, partially compensating for adaptive immune deficits in IDH1-mutant tumors [78].

In vivo, D-2HG and L-2HG, two enantiomers of 2HG administration led to a reduction in ILC1 and ILC3 counts across several tissues, with L-2HG having a broader suppressive effect. The decline in ILC subsets in mesenteric lymph nodes, colon, spleen, and small intestine suggests systemic consequences of local oncometabolite production, potentially through circulation or via induction of regulatory pathways.

Collectively, our study provides a comprehensive overview of how IDH1 mutations and associated oncometabolites remodel the innate lymphoid cell landscape in gliomas. Our data indicate that IDH1-mutant tumors are characterized by a shift toward ILC3 dominance, altered immune checkpoint receptor expression—particularly involving PD-1 and CTLA-4—and modulation of functional cytokine responses. These immunological changes appear to be driven, at least in part, by soluble metabolic factors such as D-2-hydroxyglutarate and by direct tumor–immune interactions.

From a translational perspective, these findings suggest that pharmacologic inhibition of mutant IDH1, by reducing oncometabolite-mediated immune modulation, may have the potential to influence innate immune function within the glioma microenvironment. Accordingly, future studies may consider evaluating IDH1 inhibitors either alone or in combination with immunomodulatory approaches that affect ILC activity, to better define their impact on antitumor immunity. An important consideration of the present study is that all IDH-mutant gliomas included in the cohort harbored IDH1 mutations, while no IDH2-mutant tumors were identified. This reflects the routine diagnostic workflow at our center, where IDH status is initially assessed by immunohistochemistry targeting the canonical IDH1 R132H mutation. As this approach does not detect rare non-canonical IDH1 variants or IDH2 mutations, the absence of IDH2-mutant tumors represents a methodological limitation of the study.

Despite their distinct subcellular localizations, mutant IDH1 and IDH2 enzymes share a common neomorphic activity resulting in the production of the oncometabolite 2-hydroxyglutarate, which drives widespread metabolic and epigenetic alterations in tumor cells [66, 79, 80]. Based on this shared metabolic consequence, it is conceivable that similar immunometabolic effects on innate lymphoid cell biology may also occur in IDH2-mutant gliomas. However, this possibility remains speculative and should be addressed in future studies employing sequencing-based approaches and larger, molecularly diverse cohorts.

Although our data support a role for IDH1-associated metabolites in shaping ILC phenotype, direct quantification of intratumoral D-2HG was not feasible due to limited tissue availability and the need to preserve viable cells for immunophenotyping. We therefore relied on conditioned media and controlled in vitro systems, which, while informative, do not fully recapitulate spatial metabolite gradients within the native tumor microenvironment.

An additional limitation of the present study is the use of tonsil-derived ILCs for ex vivo mechanistic experiments. While tonsillar tissue provides a practical and well-characterized source of human ILCs with sufficient yield for functional assays, these cells do not fully represent CNS-resident or tumor-infiltrating innate lymphoid populations. Tissue-specific imprinting and microenvironmental conditioning within the CNS may shape ILC phenotype in ways not fully reproduced by secondary lymphoid tissue–derived cells. Accordingly, future studies utilizing brain- or tumor-resident ILCs will be necessary to confirm the tissue-specific relevance of these findings.

Given the limited availability of IDH1-mutant glioma tissues, sample size was determined pragmatically based on patient availability during the study period. Larger and molecularly diverse cohorts will be required to validate these findings, including evaluation of biologically distinct subgroups such as mismatch repair–deficient IDH-mutant astrocytomas. This study expands our understanding of glioma immunobiology by demonstrating that innate lymphoid cells, similar to adaptive immune populations, are dynamically regulated by oncogenic mutations and associated metabolic cues. As immune checkpoint–based therapies and combination strategies involving IDH1 inhibitors continue to evolve, dissecting their effects on non–T cell compartments such as ILCs will be essential. Our findings provide a conceptual framework for future studies investigating how innate lymphoid cells contribute to antitumor immunity and how immunometabolic strategies may be rationally integrated into glioma treatment paradigms.

## Conclusions

Our study highlights that tumor-oncogenic mutations actively shape the phenotypic regulation of ILCs in the glioma microenvironment. This expanded understanding of glioma immunobiology broadens the scope of immunotherapeutic strategies and underscores the need to evaluate how interventions such as immune checkpoint blockade affect not only T cells but also these innate immune compartments. By elucidating how particular oncogenic changes hinder ILC activities and pinpointing methods to mitigate these suppressive pathways, we are facilitating the development of innovative ILC-targeted strategies. Our findings collectively provide a conceptual framework for future studies aimed at exploring strategies to modulate innate immune responses and improve antitumor immunity in these challenging malignancies.

## Supplementary Information

Below is the link to the electronic supplementary material.

Supplementary file1 (DOCX 2886 KB)

## Data Availability

The datasets used and/or analyzed during the current study are available from the corresponding author on reasonable request.

## Abbreviations

AML: Acute myeloid leukemia
C57BL/6: Common inbred mouse strain
CD127: Interleukin-7 receptor alpha chain
CFSE: Carboxyfluorescein succinimidyl ester
CML: Chronic myeloid leukemia
CTRL: Control
CTLA-4: Cytotoxic T-lymphocyte–associated protein 4
D-2HG: D-2-hydroxyglutarate
FSC-A: Forward scatter area
GBM: Glioblastoma multiforme
GM-CSF: Granulocyte-macrophage colony-stimulating factor
IDH1 Mut: Isocitrate dehydrogenase 1 mutation
IDH1 Mut-U87: U87 glioma cell line expressing IDH1 mutation
IDH-WT: Isocitrate dehydrogenase wild-type
ILC: Innate lymphoid cell
IP: Intraperitoneal
Ki-67: Proliferation marker Ki-67
L-2HG: L-2-hydroxyglutarate;
MFI: Mean fluorescence intensity
PD-1: Programmed cell death protein 1
PMA: Phorbol 12-myristate 13-acetate
R132H: Arginine-to-histidine mutation at position 132
SI: Small intestine
SSC-A: Side scatter area
TET: Ten-eleven translocation enzyme
Th: T helper
U87-MG: Human glioblastoma cell line
SEM: Standard error of the mean
α-KG: Alpha-ketoglutarate
mLN: Mesenteric lymph node
