# Supplementary material for: IDH1 Mutant Glioma Favors Group 3 Innate Lymphoid Cells and Is Resistant to Immune Checkpoint Expression
Source: Inflamm Res. 2026 Apr 1;75(1):80. doi: 10.1007/s00011-026-02223-8 (PMC13038673; doi:10.1007/s00011-026-02223-8)
Supplement: Supplementary file 1 — Supplementary file1 (DOCX 2886 KB) [file 11_2026_2223_MOESM1_ESM.docx]

**Supplementary Figure 1 – (A-E)** Flow cytometry graphs showing **(A)** TNFα, **(B)** IL 2, **(C)** IL17, **(D)** IFN-γ, **(E)** GM-CSF production by ILCs. (Bar graphs in Figure 4)

**Supplementary Figure 2 –** Effect of D2HG on apoptosis of ILCs.

**Supplementary Figure 3 –** ILC proliferation was analysed at 0-6 mg/mL doses of D-2HG. Flow cytometry representation.

**Supplementary Figure 4 –** Effect of D-2HG and L-2HG on the percentage of ILCs in mouse lymphoid non-lymphoid tissues.(Absolute number of bar graphs in Figure 7)

**Supplementary Figure 5.** Transcription factor–based validation of ILC subset identity/plasticity following ex vivo co-culture. **(A)** Representative gating strategy for sorting human tonsil-derived innate lymphoid cells (ILCs). Cells were gated on FSC/SSC, singlets, lineage-negative (CD3/CD14/CD16/CD19/CD56) and CD127⁺ populations prior to sorting. Post-sort purity analysis is shown. **(B)** Representative flow cytometry plots showing intracellular expression of T-bet (ILC1-associated) and RORγt (ILC3-associated) transcription factors in ILCs cultured alone (ILC), with IDH1-wildtype U87 glioma cells (U87-MG + ILC), or with IDH1-mutant U87 cells (IDH1Mut-U87 + ILC) for 3 days. Quadrants indicate ILC1 (T-bet⁺), ILC2 (GATA3-associated), and ILC3 (RORγt⁺) populations. **(C)** Quantitative analysis of transcription factor–defined ILC1, ILC2, and ILC3 frequencies under the indicated culture conditions. Data are presented as mean ± SEM. Statistical significance is indicated as shown (*p ≤ 0.05, **p ≤ 0.01, ***p ≤ 0.001, ****p ≤ 0.0001).


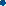

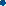

**Supplementary Figure 6.** Functional assessment of innate cytotoxic activity in short-term glioma co-culture assays. **(A)** Representative flow cytometry plots showing gating strategy and CD107α surface expression on sorted tonsil-derived ILCs and lineage-positive (NK-containing) populations following 5-hour co-culture with U87-MG (IDH1-wildtype) or IDH1Mut-U87 glioma cells. **(B–C)** Quantification of CD107α expression in lineage-positive (NK-containing) effector cells, shown as percentage of CD107α⁺ cells **(B)** and mean fluorescence intensity (MFI) **(C). (D)** Quantification of tumor cell apoptosis following NK-containing effector co-culture, assessed by Annexin V/7-AAD staining. **(E–F)** Quantification of CD107a expression in sorted ILCs following co-culture, shown as percentage of CD107α⁺ cells **(E)** and MFI **(F). (G)** Quantification of tumor cell apoptosis following ILC co-culture, assessed by Annexin V/7-AAD staining. Effector and target cells were co-cultured at an effector:target (E:T) ratio of 1:2.5 (10,000 ILCs with 4,000 glioma cells per well) for 5 hours. Tumor cells were gated separately at the end of co-culture. Experiments were independently repeated using tonsillar samples from five donors. Statistical significance is indicated as shown (*p < 0.05; **p < 0.01; ****p < 0.0001; ns, not significant).
